# Psychometric properties of the TACT framework—Determining rigor in qualitative research

**DOI:** 10.3389/frma.2023.1276446

**Published:** 2024-01-08

**Authors:** Ben Kei Daniel, Mustafa Asil, Sarah Carr

**Affiliations:** ^1^Higher Education Development Centre, University of Otago, Dunedin, New Zealand; ^2^Faculty of Health Sciences and Medicine, Bond University, Gold Coast, QLD, Australia; ^3^Otago Business School, University of Otago, Dunedin, New Zealand

**Keywords:** rigor, qualitative research, validity, confirmatory factor analysis (CFA), TACT

## Abstract

**Introduction:**

The credibility of qualitative research has long been debated, with critics emphasizing the lack of rigor and the challenges of demonstrating it. In qualitative research, rigor encompasses explicit, detailed descriptions of various research stages, including problem framing, study design, data collection, analysis, and reporting. The diversity inherent in qualitative research, originating from various beliefs and paradigms, challenges establishing universal guidelines for determining its rigor. Additionally, researchers' often unrecorded thought processes in qualitative studies further complicate the assessment of research quality.

**Methods:**

To address these concerns, this article builds on the TACT framework, which was developed to teach postgraduate students and those new to qualitative research to identify and apply rigorous principles and indicators in qualitative research. The research reported in this article focuses on creating a scale designed to evaluate the psychometric properties of the TACT framework. This involves analyzing the stability of its dimensions and understanding its effectiveness as a tool for teaching and research.

**Results:**

The study's findings indicate that the TACT framework, when assessed through the newly developed scale, exhibits stable dimensions consistent with rigorous qualitative research principles. The framework effectively guides postgraduate students and new researchers in assessing the rigor of qualitative research processes and outcomes.

**Discussion:**

The application of the TACT framework and its evaluation scale reveals several insights. Firstly, it demonstrates the framework's utility in bridging the gap in pedagogical tools for teaching rigor in qualitative research methods. Secondly, it highlights the framework's potential in providing a structured approach to undertaking qualitative research, which is essential given this field's diverse methodologies and paradigms. However, the TACT framework remains a guide to enhancing rigor in qualitative research throughout all the various phases but by no means a measure of rigor.

**Conclusion:**

In conclusion, the TACT framework and its accompanying evaluative scale represent significant steps toward standardizing and enhancing the rigor of qualitative research, particularly for postgraduate students and early career researchers. While it does not solve all challenges associated with obtaining and demonstrating rigor in qualitative research, it provides a valuable tool for assessing and ensuring research quality, thereby addressing some of the longstanding criticisms of the quality of research obtained through qualitative methods.

## Introduction and related research

The qualitative research methodology comprises several individually unique methods, with limited standardization of approaches and procedures. This lack of methodological standardization is problematic in achieving rigor (Williams et al., 2020). The scarcity of research is not surprising, considering the diverse range of approaches through which qualitative research can be conducted. Rigor, broadly defined, entails the capacity to be extremely thorough, systematic, consistent, methodical, and cautious. For several years, the literature has emphasized the value of rigor in qualitative studies (Guba and Lincoln, 1981; Morse et al., 2002; Lietz et al., 2006; Morse, 2015; Noble and Smith, 2015). For instance, Tong and Dew (2016) suggested that qualitative studies must be conducted using a rigorous approach and that the findings need to be comprehensively reported. Achieving rigor requires demonstrating that research outcomes can be applied to solve problems (Noble and Smith, 2015). It also means that the entire process of undertaking research is systematic and methodically transparent and that findings are accurately reported (Johnson et al., 2020).

Issues of rigor and relevance are likely to vary in complexity depending on the types of research questions, participants' characteristics and project size (Camfield, 2019).

Ensuring and upholding consistency in the approach, analysis, and reporting of research outcomes is of utmost importance due to the growing variety of qualitative research methods and designs (Daniel, 2019). While the significance of ensuring rigor in qualitative research methods is acknowledged (Daniel, 2018; Forero et al., 2018), a divided stance exists within the literature on practically achieving it. Some researchers have established universal criteria and standards for evaluating qualitative research grounded in interpretative ontologies (Shenton, 2004), while others have called for systematic and standardized procedures similar to those used in quantitative research (Morse et al., 2002). The adoption of general guidelines for evaluating qualitative research studies has faced criticism, as universal standards for judging qualitative research outcomes undermine the complexity and polarity of qualitative research methodology (Yardley, 2000; Dixon-Woods et al., 2004).

The complexity and plurality of methods in qualitative research can be attributed to its epistemic subjectivity, with the interpretation of data based on what is observed and how it was observed enriching the researcher's reflections and experiences of the social world (El Hussein et al., 2015; Hartman, 2015; Noble and Smith, 2015; Cypress, 2017). Barbour (2001) cautioned against using a checklist for determining rigor, asserting that improper use could reduce qualitative research to a set of technical procedures, thereby undermining the unique contribution of the systematic approach inherent to qualitative research.

Despite the diverse perspectives on achieving rigor in research presented in the literature, a universal framework is needed to provide students with clear guidance in research methods (Daniel, 2018). Such a generic framework acts as a decision-support tool, offering a comprehensive structure for novice qualitative researchers. Conducting meaningful qualitative research involves making numerous decisions, some of which are intricate and counterintuitive. This article presents the psychometric properties of the TACT framework (Daniel, 2018, 2019). TACT was designed to assist qualitative researchers in evaluating and determining the level of rigor in their studies. The framework comprises several indicators or dimensions: trust, auditability, credibility, and transferability (TACT).

## The TACT framework

**Figure 1:**
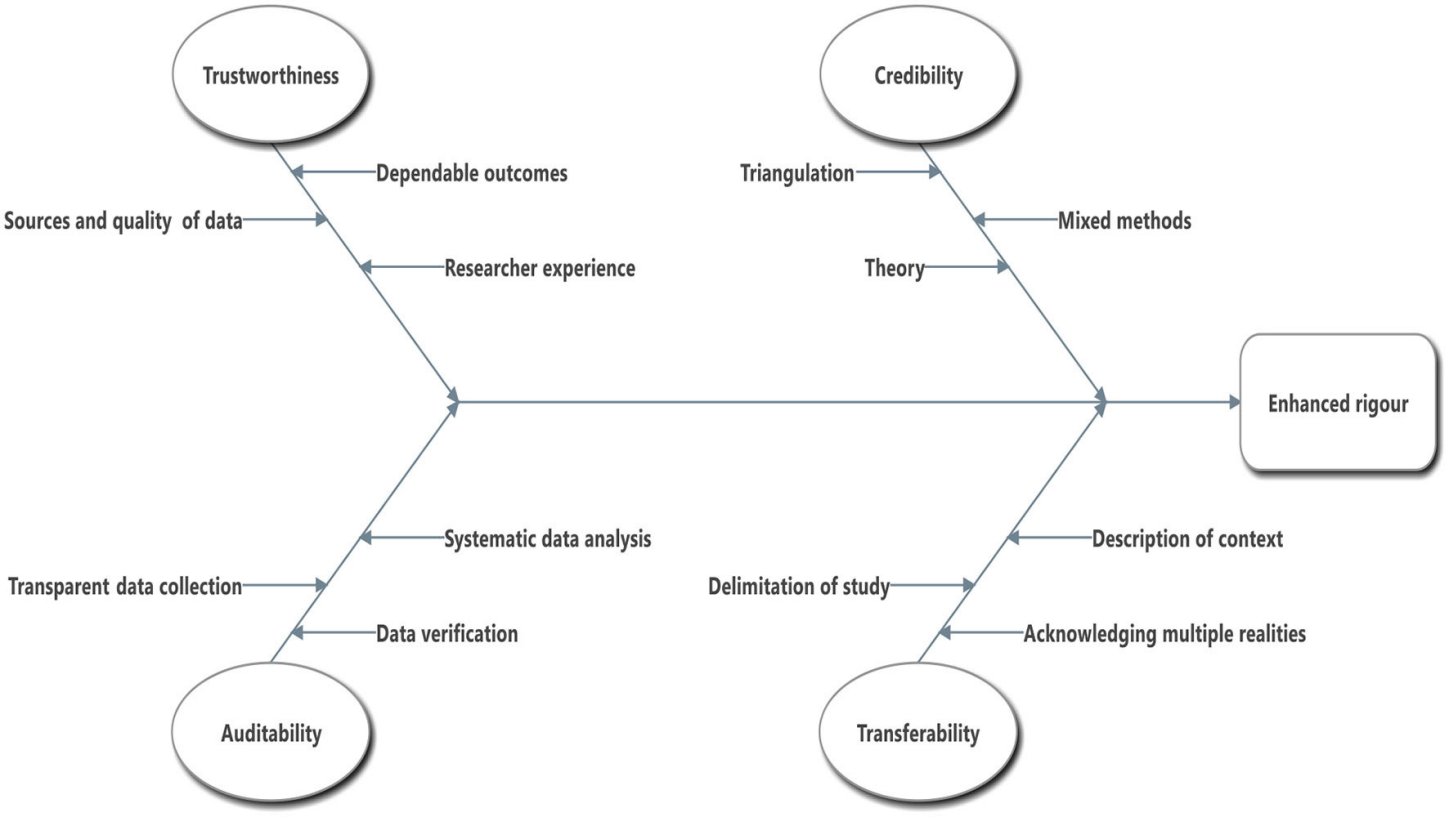
TACT model for assessing rigor in qualitative research (Daniel, 2018).

The TACT framework, which stands for trustworthiness, auditability, credibility, and transferability, offers researchers indicators to assess the rigor of qualitative research outcomes (Figure 1). These indicators were initially developed based on literature by Daniel (2018). The framework draws from other research endeavors to enhance the quality of qualitative research outcomes, such as studies by Morse et al. (2002), Koch (2006), and Johnson et al. (2020).

Trust is a fundamental aspect of ensuring the rigor of qualitative research. It has long been used to measure the truthfulness of research findings. Guba (1981) presented a model of rigor in qualitative research centered around trustworthiness: truth value, applicability, consistency, and neutrality. However, applying this model can be complex and time-consuming, requiring various strategies and situating findings within participants' views (Lietz et al., 2006; Sinkovics and Ghauri, 2008). Reflexivity, the researcher's self-awareness of their thoughts and actions in different contexts, can contribute to achieving trustworthiness (Meyrick, 2006).

### Trustworthiness

Trust is a fundamental element for meaningfully interpreting qualitative research outcomes. It involves demonstrating relevance and confidence in research findings and establishing the authenticity of the results. Researchers achieve trustworthiness through a systematic and methodical approach, demonstrating data analysis and interpretation consistency. Trust is also essential in maintaining the overall integrity of research outcomes. This integrity in the qualitative research process ensures trustworthiness without advocating for a singular approach or disregarding opposing viewpoints.

### Auditability

Auditability refers to a systematic recording of the research process, commonly known as an “audit trail.” Researchers present a clear pathway of decisions made during the research, facilitating reflection on the process. Auditability includes external and internal aspects. External auditability allows end-users to review research findings, while internal auditability involves scrutinizing methodological integrity concerning research questions, design, analysis, and conclusions.

### Credibility

Credibility is crucial in all qualitative research outcomes. It involves demonstrating the ultimate truth in research conclusions and validating the authenticity and reliability of the findings. Researchers achieve credibility by ensuring that research design, data collection methods, analysis, and reporting align coherently with the research outcomes. Triangulation, member checking, peer debriefing, and prolonged engagement with participants are ways researchers can use to enhance credibility.

### Transferability

Transferability enables researchers to apply research outcomes from one qualitative study to other similar settings or groups of people, offering valuable lessons. To achieve transferability, researchers must describe the study context and sample characteristics. Expert knowledge of participants and their understanding of the phenomenon under study are critical factors in the recruitment and selection of the sample. Detailed descriptions of real-life settings and participants' worldviews contribute to achieving transferability.

In summary, the TACT framework provides valuable indicators to assess the rigor of qualitative research outcomes, encompassing trustworthiness, auditability, credibility, and transferability. Researchers can use these indicators to enhance the quality and applicability of their qualitative research findings.

## Purpose of the study

This study explores factors associated with achieving rigor in qualitative research studies, identifying the strategies needed to support them. The study is part of a large research programme looking at various ways to support the pedagogy of research methods.

The primary purpose of this study was to develop a comprehensive measure for assessing rigor in qualitative research studies and provide validity and reliability evidence to support its use for research purposes. We also wanted to examine the relationship between TACT constructs and some selected demographic factors. This study attempts to answer the following research questions:

1. How do we develop a measure of rigor in qualitative research?
2. To what extent is there evidence to support the factorial validity of the TACT (trustworthiness, auditability, credibility and transferability)?
3. To what extent are TACT factors associated with degree and stages in a postgraduate programme, academic division, and methodology experience?

## Method and materials

### Participants

We invited participants to participate in this study through an online questionnaire deployed for 2 years. The participants were 434 researchers at a research-intensive public university in New Zealand. Participants were enrolled in workshops on advanced topics in qualitative research methods taught by one of the authors. Of the participants, 49.5% (*n* = 215) were PhD, 26.3% were Masters (*n* = 114), 1.9% were diploma (*n* = 8) holders, and 21.0% were staff members (*n* = 91). Only six participants did not report their degree programme. Representation by academic division was 30.9% humanities (*n* = 134), 26.0% health science (*n* = 113), 23.3% commerce (*n* = 101), 7.8% science (*n* = 34), and 9.4% interdisciplinary (*n* = 41). Of the participants, 11 did not provide their academic division.

Concerning the status of research, 33.2% (*n* = 144) were planning research, 30.9% (*n* = 134) were writing up a thesis, and 27.4% (*n* = 119) were doing research. The remaining 7.8% (*n* = 34) were either making amendments to the thesis, awaiting graduation or not a student/staff member. Three participants did not report their stages in the programme. Regarding self-reported experience (feeling comfortable) with methodology, 30.6% were qualitative, 27.0% were mixed methods, 24.7% were quantitative, 5.3 % were all three traditions, 2.3% were qualitative and quantitative, and 3.2% did not provide information on the methodological experience.

### Instrument development

The four dimensions of TACT were identified from various discourses of rigor in the literature on qualitative research methods. The verification of the TACT started with the development of a rating tool. The initial TACT scale contained 16 items to assess four dimensions of rigor in qualitative research studies: trustworthiness, auditability, credibility and transferability. Participants rated each item on a 5-point Likert scale, where 1 = *very important*, 2 = *important*, 3 = *neutral*, 4 = *less critical*, and 5 = *not important*. For ease of understanding, the items in the present study were reverse-coded with higher scores indicative of greater importance.

In this study, we examined the psychometric characteristics of the TACT scale using a Structural Equation Modeling (SEM) approach to provide validity and reliability evidence for its use with tertiary education researchers. The items in the TACT scale are shown in the Appendix.

### Summary of statistical analyses

We conducted the data analyses in three stages. First, we checked the data for outliers and missing cases. Second, we adopted a Structural Equation Modeling (SEM) approach, focusing on Confirmatory Factor Analysis (CFA), to evaluate the factorial structure of the TACT scale using the MPlus 7 software (Muthén and Muthén, 2012). The hypothesized model was compared to other alternative (competing) models. The convergent and discriminant validities of the data were then established.

In the third stage, we utilized univariate MANOVA to explore potential significant mean differences in TACT components across participants' degree and status in the programme, academic divisions, and the methodology experience. An alpha level of 0.05 was used as a guideline for determining the significant effects of variables. The *p*-values for the univariate analyses were corrected relative to the number of subscales (e.g., the *p*-value for significance = 0.05/number of subscales). We also reported effect size measures, specifically partial eta squared (η^2^), to indicate the magnitude of the significant differences. The small, medium and large effects correspond to values of η^2^ of 0.01, 0.06, and 0.14 (Richardson, 2011).

The Weighted Least Squares Mean and Variance Adjusted (WLSMV) method was chosen as the Confirmatory Factor Analysis (CFA) estimator due to the data's ordered-categorical nature. Lubke and Muthén (2004) demonstrated that treating ordinal data as continuous can lead to inaccurate outcomes. WLSMV, a robust estimation technique, is recommended for modeling ordinal data (Flora and Curran, 2004; Brown, 2006). Robust estimation techniques are instrumental and effective when dealing with non-normal data distributions (Finney and DiStefano, 2006) because they apply a scaling factor to account for non-normality (Muthén and Muthén, 2006).

As suggested in the literature, we used several different goodness of fit indices to compare the different models and evaluate model-data fit (Cheung and Rensvold, 2002; Fan and Sivo, 2005, 2007). Each of these indices reflects a different aspect of model fit and may not be equally sensitive to different model conditions (Fan and Sivo, 2007). Therefore, it is essential to use multiple indices rather than relying on a single measure (Hair et al., 2010).

We evaluated model fit using the Root Mean Square Error of Approximation (RMSEA), the Comparative Fit Index (CFI), and the Tucker-Lewis index (TLI). In contrast, we reported the chi-square (χ^2^) values, which were not used for model fit decisions due to their sensitivity to sample size, model complexity, and distribution of variables. Based on the literature, the criteria we used for “acceptable” or “good” fit (Browne and Cudeck, 1992; MacCallum et al., 1996; Hu and Bentler, 1998; Hair et al., 2010) included: a non-significant chi-square (χ^2^), RMSEA with values < 0.08 indicating an acceptable fit and values < 0.05 indicating a good fit, and CFI and TLI with values >0.90 being indicative of reasonable fit and values >0.95 indicating a good fit.

After assessing the model fit as part of factorial validation, we further examined item loadings, factor correlations and reliabilities to provide evidence for convergent and discriminant validity evidence. The limitations of coefficient alpha (α) as a measure of reliability estimate are well-reported in the literature (Sijtsma, 2009; Teo and Fan, 2013). Therefore, we calculated and reported McDonald's (1999) omega (ω) for each TACT dimension as a better reliability estimate.

## Results

### Descriptive statistics

We have not identified any univariate outliers that have an effect on the results. The proportion of missing cases for each TACT item was minimal, ranging from mostly zero to 2%. Rather than deleting, the Expectation Maximization (EM) algorithm was utilized to impute the missing cases. The means and standard deviations for the four factors of the TACT scale are summarized in Table 1.

**Table 1:** Descriptive statistics of TACT factors.

	**Number of items**	***M***	***SD***	***Min***	***Max***
Trustworthiness (TW)	3	4.36	0.69	2.00	5.00
Auditability (AU)	4	4.37	0.59	2.50	5.00
Credibility (CR)	4	3.89	0.72	2.00	5.00
Transferability (TR)	4	4.45	0.55	2.00	5.00

Our results indicated that factor means ranged from 3.89 to 4.37, suggesting that most participants endorsed the statements as “important” or “very important.” The standard deviations ranged from 0.55 to 0.72, indicating that the dispersion of responses for each factor was somewhat similar. However, examining the item means, ranging from 1.00 to 5.00, revealed that some researchers ranked the statements as “not important” or “less important.” For example, 65 (15%) researchers surprisingly reported that “ensuring the outcomes of a qualitative research study can be verified by theory/literature” was unnecessary or less essential for them.

### Confirmatory factor analysis (CFA)

To provide validity evidence for the internal factor structure of the TACT measure, we tested and compared the goodness-of-fit of different competing models as suggested in the literature (Noar, 2003; Strauss and Smith, 2009). The hypothesized four-factor correlated TACT model was compared to two other alternative competing models. The alternative models included: (a) a one-factor (unidimensional) model that assumed all manifest variables loaded on a single factor and (b) a four-factor uncorrelated (orthogonal) model that suggests all the factors in the model are unrelated.

Support for the one-factor model means that we are measuring a unidimensional construct, and researchers are not differentiating the factors that assess rigor in qualitative research. Evidence for the four-factor orthogonal model indicates that TACT factors are distinct and independent. Support for the hypothesized four-factor correlated (oblique) model would suggest that researchers discern between four TACT factors related to each other. The goodness-of-fit measures of hypothesized and alternative models are summarized in Table 2.

**Table 2:** Confirmatory factor analysis of alternative models.

**Model**	**χ^2^**	***df***	**RMSEA**	**RMSEA 90% CI**	**CFI**	**TLI**
One-factor	1,168.32	104	0.15	0.15, 0.16	0.65	0.59
Four-factor uncorrelated	2,021.50	104	0.21	0.20, 0.21	0.36	0.26
Four-factor correlated	861.45	98	0.13	0.12, 0.14	0.75	0.69
Four-factor correlated (modified)	313.92	82	0.08	0.07, 0.09	0.92	0.90

RMSEA, Root Mean Square Error of Approximation; CFI, Comparative Fit Index; TLI, Tucker-Lewis Index.

^*^p < 0.01.

As evident from Table 2, the results of the unidimensional and four-factor uncorrelated models revealed that these models did not represent the sample data sufficiently. The RMSEA, CFI, and TLI values did not meet the commonly acceptable fit criteria. The fit of the hypothesized four-factor correlated model (RMSEA = 0.13; CFI = 0.75; TLI = 0.69) was neither adequate. To pinpoint the sources of misfit, we further examined factor loadings, residual matrices and modification indices. We found that the factor loading for the second trustworthiness item was relatively low (λ = 0.29).

A closer examination of the wording of this item, “Ensuring research outcomes conform to research's assumptions or a well-established theory or both,” yielded that it was the only double-barrelled item, which was probably cognitively challenging to the respondents, so the item was removed from the scale. Examination of the modification indices suggested that incorporating residual covariance into the model would result in a more accurate fit. The most substantial modification indices were noted between items TW3 and TW4 and between items CR3 and CR4. Applying these modifications resulted in a significant improvement in model fit. The results revealed an acceptable model fit for the modified four-factor model (RMSEA = 0.08; CFI = 0.92; TLI = 0.90). Standardized factor loadings and reliabilities for the revised model are presented in Table 3.

**Table 3:** Results for the measurement model.

**Item**	**Standardized factor loading**	**Reliability omega (ω)**
**Trustworthiness (TW)**		0.77
TW1	0.75	
TW3	0.59	
TW4	0.58	
**Auditability (AU)**		0.80
AU1	0.73	
AU2	0.61	
AU3	0.82	
AU4	0.71	
**Credibility (CR)**		0.70
CR1	0.56	
CR2	0.68	
CR3	0.50	
CR4	0.63	
**Transferability (TR)**		0.77
TR1	0.74	
TR2	0.60	
TR3	0.63	
TR4	0.81	

Standardized factor loadings for the hypothesized model were significant and ranged from 0.50 to 0.82, supporting convergent validity. All of the items were strong indicators of the factors they were related to. The omega reliability estimates exceeded the recommended level (0.70). Factor correlations are presented in Table 4.

**Table 4:** TACT factor correlations.

	**TW**	**AU**	**CR**
Trustworthiness (TW)	-		
Auditability (AU)	0.86	-	
Credibility (CR)	0.23	0.50	-
Transferability (TR)	0.49	0.84	0.40

The correlations among the TACT factors ranged from 0.23 to 0.86, indicating the presence of discriminant validity evidence. The strongest correlations were observed between trustworthiness—auditability and transferability—auditability. Additionally, we noted modest correlations among other pairs of factors. The results from CFA analyses and reliability estimates supported the validity evidence at both the item and construct levels of the TACT scale. Thus, the proposed research model's constructs are deemed suitable for further analyses.

### Differences on TACT

A multivariate analysis of variance (MANOVA) was employed to determine whether significant differences exist in TACT components about researchers' degree and status in the programme, academic division, and methodology experience. Dependent variables were the researchers' mean scores on each TACT subscale. Independent variables were a degree in the programme (masters, PhD, and staff), stages in the programme (planning research, doing research, and writing a thesis), academic division (commerce, humanities, and health sciences), and the methodology experience (qualitative, quantitative, and mixed methods). We omitted groups with small sample sizes from the analyses. MANOVA results revealed significant differences (Wilks' Lambda < 0.001) in the dimensions of the TACT based on researchers' degree and status in the programme, academic division, and methodology experience. Further univariate and *post-hoc* comparisons (with Bonferroni) were utilized and summarized in Table 5.

**Table 5:** Differences in the TACT subscales.

**Group**	**TACT subscale**	***F***	***p***	**Partial eta squared**	***Post-hoc*** **(high-low)**		
Degree	Trustworthiness	9.72	< 0.001	0.05	Staff—Masters	PhD—Masters	
	Auditability	9.86	< 0.001	0.05	Masters—PhD		
	Credibility	10.53	< 0.001	0.05	Masters—PhD	Masters—Staff	
	Transferability	3.15	0.04				
Status	Trustworthiness	29.27	< 0.001	0.13	Doing—Planning	Writing—Planning	
	Auditability	1.53	0.22				
	Credibility	13.89	< 0.001	0.07	Planning—Doing	Planning—Writing	
	Transferability	0.01	0.99				
Division	Trustworthiness	4.75	0.01	0.03	Health—Commerce		
	Auditability	21.06	< 0.001	0.11	Humanities—Commerce	Health—Commerce	Humanities—Health
	Credibility	21.16	< 0.001	0.11	Humanities—Health	Humanities—Commerce	Health—Commerce
	Transferability	3.01	0.05				
Methodology	Trustworthiness	13.96	< 0.001	0.07	Qualitative—Mixed	Quantitative—Mixed	
	Auditability	2.56	0.08				
	Credibility	0.61	0.54				
	Transferability	3.62	0.03				

P < 0.0125 (Bonferroni corrected).

#### Degree differences

Significant variations were observed across the TACT subscales based on participants' degree affiliations. Noteworthy disparities were evident in the “Trustworthiness,” “Auditability,” and “Credibility” dimensions. *Post-hoc* comparisons indicated that participants holding PhD, Master, and Staff positions rated these factors differently, emphasizing the role of academic qualifications in shaping perceptions of research rigor.

#### Status distinctions

Exploring differences in research status revealed substantial discrepancies in the appraisal of “Trustworthiness” and “Credibility” subscales. Participants engaged in distinct research phases, encompassing “Doing,” “Planning,” and “Writing,” exhibited divergent evaluations of these dimensions. These findings illuminate the dynamic interplay between research activities and the perceived rigor of research endeavors.

#### Divisional variances

The academic division to which participants belonged yielded meaningful variations in the assessment of “Auditability” and “Credibility” dimensions. Participants affiliated with different academic domains demonstrated divergent ratings, underscoring the influence of disciplinary perspectives on appraisals of research rigor.

#### Methodological perspectives

Participants' research methodologies engendered significant disparities in evaluating the “Trustworthiness” and “Transferability” subscales. Notable differences were observed between qualitative, mixed, and quantitative research practitioners. These findings reflect the nuanced interrelationships between research paradigms and perceptions of research rigor.

## Discussion and conclusion

The most challenging aspects of conducting high-quality research include making sense of voluminous data, imposing order, structure and meaning, identifying helpful information, making it logical, sensible and meaningful, and assessing the quality of qualitative research outcomes. In response to these challenges, this study aimed to develop a comprehensive measure to assess rigor in qualitative research studies and provide validity evidence to support its use for research purposes. By doing so, this research contributes to the advancement of rigor within the qualitative research domain. Notably, employing confirmatory factor analysis (CFA), the study establishes empirical support for the multidimensional structure of the TACT, which comprises four factors: trustworthiness, auditability, credibility, and transferability.

The magnitude of the factor loadings demonstrated that items were strong indicators of their respective dimensions within the TACT framework. The observed correlations among factors were in the expected direction, further providing additional validity evidence. The computed omega reliability estimates surpass the established threshold of 0.70, indicative of the robust internal consistency of the measured constructs. These findings collectively endorse the TACT as a valuable measure for assessing rigor in qualitative research. To our knowledge, this is the first analytical tool developed explicitly to measure the level of rigor in qualitative research.

The MANOVA findings shed light on the intricate interconnectedness among academic credentials, research status, academic disciplines, and research methodologies, collectively enriching the diverse spectrum of perspectives regarding research rigor.

The TACT framework equips researchers with informative indicators to navigate the assessment of rigor. While not rigid, these indicators manifest as theoretical and empirically grounded guidelines. Qualitative research methods are used to explore complex social phenomena. The lack of standardization in these methods and the challenge of replicating findings have led to growing criticism (Barbour, 2001; Filep et al., 2018). Notably, these critiques have described qualitative methods as biased and limited in generalizability (Cope, 2014; Hayashi et al., 2019).

### Implications to pedagogy and research

Teaching rigor in qualitative research presents challenges partly because rigor, like many other social phenomena in qualitative research, is highly abstract and constructed, with meanings that can differ from one person to another. By identifying and validating its constituent dimensions, teachers of research methods can provide students with concrete elements that they can critically inspect and discuss, and teachers can emphasize the importance of maintaining rigor throughout the research process. Teachers can use the TACT framework to show students how to integrate rigor when framing research questions, designing studies, collecting and analyzing data, and reporting findings clearly and precisely. The framework also highlights the importance of reflexivity in qualitative research by providing students with clear indicators they should think critically about in their research design and decision-making. This active reflection will help students justify their approach at each research stage.

Introducing the TACT framework as an anchor of rigor in qualitative research can lead to higher quality and credibility. Researchers well-versed in rigorous qualitative methods are better equipped to produce trustworthy findings that contribute meaningfully to their field of study. By upholding rigorous research practices, the validity and reliability of qualitative research can be enhanced, thus bolstering the confidence of scholars, policymakers, and practitioners in the outcomes. Additionally, a focus on rigor in qualitative research can pave the way for more transparent and replicable research practices, fostering a culture of accountability and openness within the research community. This, in turn, promotes a positive impact on advancing knowledge and addressing complex social and cultural phenomena robustly and meaningfully.

### Suggestions for future research

The four dimensions of rigor in the TACT framework are not obligatory benchmarks of rigor but rather serve as important indicators that guide researchers in enhancing the quality of research outcomes. These dimensions encourage researchers to consider thoughtful strategies to enhance research quality by addressing one or more of these facets. Further validation and replication studies involving the TACT measure remain necessary to substantiate its stability, reliability and construct validity. We believe conducting measurement invariance analyses across various settings or time points holds significant potential for fruitful research. Researchers may also be interested in examining the extent to which scores on the TACT measure are associated with gender, ethnicity or the age of the researchers.

Although the four-factor model provided the best fit, we acknowledge that there remains potential for enhancing the psychometric properties of the TACT scale. In this study, we tried to model and interpret a second-order (higher-order) factor model with the four scale factors subordinating to a single second-order factor. However, we were unable to assess the goodness-of-fit of this model and compare it with other models due to the covariance matrix not being positive definite, resulting in an inadmissible solution. Thus, we encourage researchers to consider and test a higher-order factor model which could potentially encapsulate a broader and more overarching conceptualization of the rigor construct.

In the future, we will explore whether the TACT framework can be applied to domains in qualitative research that often do not neatly fall into the steps by logical sequencing of qualitative research such as researching the unconscious and indigenous research. Central to this exploration is applying TACT's key elements, such as Auditability, to test whether integrating this element to every stage of the research process, including problem definition, design, methods, and outcomes, can improve the quality of research findings, fosters more profound understanding and constructive dialogue among scholars and stakeholders.

In the context of Indigenous Research Methods (IRM) with a focus on decolonization, it would be worthwhile to use the TACT framework in the entire research cycle and analyze whether aspects such as Trustworthiness and Auditability can empower historically marginalized communities to understand and trust the process and outcome of the research. We believe that applying TACT across various stages of research, from question formulation to presentation, encourages the inclusion of colonized communities' perspectives, making them partners in the research instead of subjects being researched and exploited. It is possible that such an approach shifts from traditional, top-down methods toward a more collaborative, culturally sensitive, and transparent model. Furthermore, the psychometric validation of the TACT tool shows that its integrity makes it a suitable guide for researchers within decolonized epistemologies and ontologies, fostering a significant paradigm shift toward more inclusive, respectful research methodologies.

### Limitations

A notable limitation of this study is the use of self-report instruments for data collection, which introduces the possibility of inherent bias. Furthermore, a constraint pertains to the sample composition. Since we utilized a convenience and voluntary sampling approach, our sample may not comprehensively represent the entire population. Consequently, we advocate for future investigations to employ the TACT measure with larger sample sizes, thereby enhancing the generalizability of findings.

We acknowledge that the TACT framework presented in this article is quantitatively oriented and structured, which may limit its ability to adequately capture the intricacies of areas such as researching the unconscious. Researching into the unconscious is predominantly qualitative and fluid, marked by complexities that do not neatly fit within the rigid parameters of quantitative methods or logical steps in qualitative methods. In structured and numerical analysis, frameworks such as TACT may not adequately grasp the full depth and breadth of the unconscious, an area that frequently defies conventional structured or logical qualitative research approaches. However, TACT can still help researchers in the unconscious domains document their procedures, approaches, and rationales in all stages of the research process. They can use it as a reflective tool.

Further, it is worth noting that researching the unconscious is influenced by myriad factors that are generally not quantifiable. These include cultural influences, psychological dynamics, and social elements, collectively weave a complex tapestry defining the unconscious. To understand this aspect of human cognition, we require an approach capable of navigating these diverse and often subtle influences that may not lend themselves to straightforward quantitative measurement or strict structural frameworks. This necessitates a more flexible and nuanced method of studying the unconscious, which truly appreciates the rich and varied dimensions contributing to its formation.

## Data availability statement

The raw data supporting the conclusions of this article will be made available by the authors, without undue reservation.

## Ethics statement

The studies involving humans were approved by University of Otago Human Ethics. The studies were conducted in accordance with the local legislation and institutional requirements. The participants provided their written informed consent to participate in this study.

## Author contributions

BD: Conceptualization, Data curation, Methodology, Validation, Writing – original draft, Writing – review & editing. MA: Formal analysis, Methodology, Project administration, Writing – original draft, Writing – review & editing. SC: Investigation, Methodology, Resources, Writing – review & editing.
