# Supplementary material for: Psychometric properties of the TACT framework—Determining rigor in qualitative research
Source: Front Res Metr Anal. 2024 Jan 8;8:1276446. doi: 10.3389/frma.2023.1276446 (PMC10800612; doi:10.3389/frma.2023.1276446)
Supplement: Supplementary file 1 [file Table_1.docx]

**APPENDIX**

**Table A1**

**TACT Scale Items**

| **Trustworthiness (TW)** | |
| --- | --- |
| TW1 | Providing adequate information for other researchers to enable them assess the quality of qualitative research |
| TW2* | Ensure that the research outcome confirms to the researcher's assumptions or a well-established theory or both. |
| TW3 | Explicitly stating one's biases in the process of undertaking the research. |
| TW4 | Clearly stating the limitations of an approach employed in the research project. |
|  |  |
| **Auditability (AU)** | |
| AU1 | Providing a thorough description of the process of undertaking the qualitative research |
| AU2 | Acknowledging that the outcomes of qualitative research findings are subject to multiple interpretations |
| AU3 | Clearly describing how data analysis is undertaken in the research project. |
| AU4 | Clearly describing the researcher's contribution to the data during the process of data collection. |
|  |  |
| **Credibility (CR)** | |
| CR1 | Clearly describing one's experience relative to the research problem. |
| CR2 | Making sure that others will have full confidence in the findings reported |
| CR3 | Ensuring the outcomes of a qualitative research study can be verified by theory/literature |
| CR4 | Making sure that findings resulting from different sets of data collected around the same problem is likely to validate each other |
| **Transferability (TR)** | |
| TR1 | Providing a detailed description of the phenomenon being studied. |
| TR2 | Clearly describing the context in which the research is undertaken. |
| TR3 | Clear description of participants (e.g. their assumptions, views, values, and thoughts). |
| TR4 | Acknowledging that participants can see the same problem in different ways. |
| ****Note*: TW2 was excluded from the final scale** | |
